# The furculae of the dromaeosaurid dinosaur *Dakotaraptor steini* are trionychid turtle entoplastra

**DOI:** 10.7717/peerj.1691

**Published:** 2016-02-09

**Authors:** Victoria M. Arbour, Lindsay E. Zanno, Derek W. Larson, David C. Evans, Hans-Dieter Sues

**Affiliations:** 1 Paleontology Research Lab, North Carolina Museum of Natural Sciences, Raleigh, NC, United States; 2 Department of Biological Sciences, North Carolina State University, Raleigh, NC, United States; 3 Department of Ecology and Evolutionary Biology, University of Toronto, Toronto, Ontario, Canada; 4 Department of Natural History, Royal Ontario Museum, Toronto, Ontario, Canada; 5 Department of Paleobiology, National Museum of Natural History, Washington, DC, United States

**Keywords:** Trionychidae, Dromaeosauridae, Theropoda, Testudines, Axestemys, Cretaceous, Maastrichtian, Hell Creek Formation, South Dakota

## Abstract

*Dakotaraptor steini* is a recently described dromaeosaurid dinosaur from the Upper Cretaceous (Maastrichtian) Hell Creek Formation of South Dakota. Included within the *D. steini* hypodigm are three elements originally identified as furculae, one of which was made part of the holotype specimen. We show that the elements described as *D. steini* ‘furculae’ are not theropod dinosaur furculae, but are rather trionychid turtle entoplastra referable to cf. *Axestemys splendida*. The hypodigm of *D. steini* should be adjusted accordingly.

*Dakotaraptor steini* DePalma et al., 2015 is a recently described dromaeosaurid dinosaur from the Upper Cretaceous (Maastrichtian) Hell Creek Formation of South Dakota. The holotype (PBMNH P.10.113.T) is given as an associated skeleton derived from a bonebed that purportedly contains the fossilized remains of other vertebrates including mammals, fish, amphibians, pterosaurs, reptiles, and birds (DePalma, 2010; DePalma et al., 2015). Included within the *D*. *steini* hypodigm are three elements that DePalma and colleagues (2015) identify as furculae: one which is part of the holotype specimen and two referred specimens—NCSM 13170 and KUVP 152429 (which was not figured, and which we have not observed directly). The furcula of PBMNH P.10.113.T was intermingled with the other elements assigned to the holotype, KUVP 152429 was found nine metres away from the holotype in the same bonebed, and NCSM 13170 was discovered as an isolated element sixteen miles from the holotype (DePalma et al., 2015). Here we demonstrate that the elements described as *D*. *steini* ‘furculae’ are not theropod dinosaur furculae, but are trionychid turtle entoplastra.

The furcula is a median, unpaired element present in extant birds and their non-avian theropod relatives (Nesbitt et al., 2009). Although the furcula is generally thought to have arisen through fusion of the clavicles, recent developmental studies suggest that the furcula is homologous with the interclavicle of early tetrapods (Vickaryous & Hall, 2010). DePalma et al. (2015) themselves noted several differences between the putative ‘furculae’ of *Dakotaraptor steini* and those of other non-avian theropod dinosaurs. We note that in PBMNH P.10.113.T and NCSM 13170, the ‘furcula’ is extremely craniocaudally compressed, and possesses flattened rami that bulge halfway along the length of the ramus, terminally asymmetrical ‘epicleidia’ with longitudinal striations, a medial juncture bearing a ventral tab (previously identified as the ‘hypocleidium’), and transversely straight, rather than caudally bowed rami. Taken together this suite of characteristics is unknown in other theropod furculae (Nesbitt et al., 2009), yet is consistent with the structure of the entoplastron in trionychid (soft-shelled) turtles.

The entoplastron is a median, unpaired element in the plastron, and, like the furcula of theropods, is a homolog of the interclavicle (Gilbert et al., 2001). In many turtle clades, the entoplastron is a roughly diamond-shaped element; however, in trionychids it takes on a flattened, slender, V-shaped to boomerang-shaped appearance, with lateral projections that diverge at roughly 90°(Hay, 1908; Vitek, 2012; Hutchison, 2013; Vitek & Joyce, 2015), reminiscent of the shape of non-avian theropod furculae. In their description of the associated fauna DePalma et al. (2015) note that multiple turtles, including trionychids (as *Trionyx* sp.) are preserved at the holotype locality (DePalma, 2010). Trionychids are common elements of Campanian-Maastrichtian North American ecosystems (Brinkman, 2003) and at least five species are represented in the Hell Creek Formation from which *D*. *steini* derives (Holroyd & Hutchison, 2002; Holroyd, Wilson & Hutchison, 2014; Vitek & Joyce, 2015). Moreover, several Campanian to modern trionychine trionychids (terminology following Hummel, 1928) have entoplastra that closely match the morphology of NCSM 13170 and the element figured as a ‘furcula’ in PBMNH P.10.113.T. Although KUVP 15249 was not examined by us or figured in the original description, DePalma et al. (2015: p. 6) considered it “virtually indistinguishable” from and “identical” to the holotype ‘furcula’, and therefore it is reasonable to assume that KUVP 15249 may also be a trionychid entoplastron.

**Figure 1:**
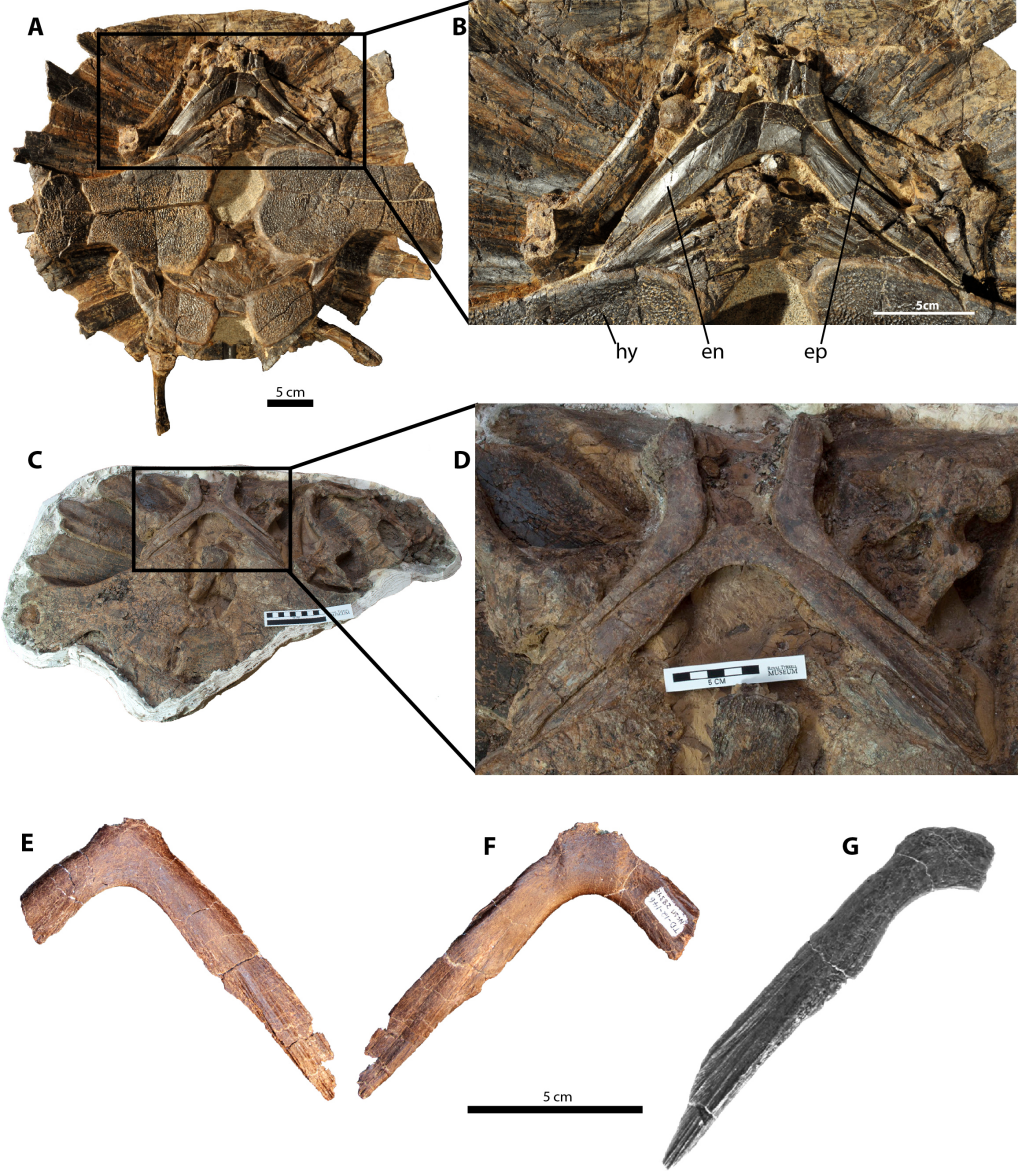
Purported furculae for the holotype and referred specimens of *Dakotaraptor steini* compared with the entoplastron of the trionychid turtle *Axestemys splendida*; anterior is up. (A–D), *Axestemys splendida* plastra in ventral view, showing the entoplastron in articulation with the other elements of the plastron. (A) and (B) ROM 1430; (C) and (D) TMP 2015.012.0011. NCSM 13170 trionychid entoplastron (referred to *D. steini* by DePalma et al., 2015) in (E) dorsal and (F) ventral views. (G) PBMNH P.10.113.T (‘furcula’ comprising part of the holotype for *D. steini*, adapted from DePalma et al., 2015). Abbreviations: hy, hypoplastron; en, entoplastron; ep, epiplastron.

Among extinct North American trionychines, the taxonomic identity of the entoplastral elements within the *D*. *steini* hypodigm can be refined on the basis of comparative morphology and relative size (Vitek, 2012). Here we follow the trionychid taxonomy of Danilov et al. (2014), but see Vitek & Joyce (2015) for a differing opinion. NCSM 13170 and PBMNH P.10.113.T exhibit an overall gracile morphology (narrow craniocaudally relative to the length of the lateral projections) as in *Axestemys splendida* (Gardner, Russell & Brinkman, 1995), other species of *Axestemys* (Vitek, 2012), *Aspideretoides allani* (Gardner, Russell & Brinkman, 1995), and *Apalone* and relatives (Vitek, 2011; Danilov et al., 2014).

Several discrete features of NCSM 13170 and PBMNH P.10.113.T are shared with select trionychid species. In NCSM 13170 the craniomedial margin of the rami junction is broad and cranially convex, bearing distinct lateral notches for contact with the epiplastra (Fig. 1). This differs from the condition seen in *Axestemys montinsana* (Vitek, 2012), yet closely matches the morphology seen in *Axestemys splendida* and other Late Cretaceous trionychids (Gardner, Russell & Brinkman, 1995). The distalmost one quarter of the ramus in PBMNH P.10.113.T (and in NCSM 13170, although the tip of the ramus is damaged) abruptly tapers asymetrically, representing the end of the contact between the entoplastron and epiplastron (Fig. 1). This morphology is identical to that seen in *Axestemys splendida* (Gardner, Russell & Brinkman, 1995; Fig. 1), *Axestemys montinsana* (Vitek, 2012) and possibly *Gobiapalone breviplastra* (Danilov et al., 2014).

The caudal margins of the rami in NCSM 13170 and PBMNH P.10.113.T bear a notch for the reception of the hyoplastron, which articulates with approximately two-thirds of the entoplastron ramus. The extent of this contact is similar in *Axestemys splendida* (Gardner, Russell & Brinkman, 1995; Fig. 1), *Axestemys montinsana* (Vitek, 2012), *Axestemys cerevisia* (Vitek, 2012), *Aspideretoides allani* (Gardner, Russell & Brinkman, 1995), and *Apalone* (Vitek, 2012), yet differs in *Aspideretoides foveatus* (Gardner, Russell & Brinkman, 1995), *Oliveremys uintaensis* (Vitek, 2011), *Gobiapalone breviplastra*, and *Gobiapalone orlovi* (Danilov et al., 2014). It is noted by Vitek (2012) that this contact in *Axestemys* is not as extensive as in *Apalone* and that *Axestemys* lacks a hyoplastral shoulder locking the entoplastron in place.

Finally, a distinctive longitudinal fluting along the distal third of each ramus for the attachment of connective tissue mars the rami in NCSM 13170 and PBMNH P.10.113.T. This is also present in *Axestemys splendida* (Campanian-Maastrichtian, Fig. 1), *Axestemys montinsana* (Paleocene; Vitek, 2012: Fig. 17), and *Oliveremys uintaensis* (Vitek, 2011).

The largest of the three trionychid entoplastra comprising the *D*. *steini* hypodigm (PBMNH P.10.113.T) pertains to a carapace approximately 60 cm in length based on comparisons with comparable materials (Figs. 1A–1D). This is consistent with the size range of *Axestemys* (Vitek, 2012), and of similar proportions to large trionychid shells known from the Hell Creek Formation (Hutchison & Archibald, 1986).

Taken together, the morphology and size of the PBMNH P.10.113.T “furcula” and NCSM 13170 indicate that they should not be referred to *Dakotaraptor steini*, and are instead most confidently identified as cf. *Axestemys splendida*. The holotype material of *Axestemys splendida* is Campanian in age, yet several specimens from the Late Maastrichtian have been referred to this taxon (Vitek, 2012; Vitek & Joyce, 2015) or are otherwise not identified to species (Holroyd, Wilson & Hutchison, 2014), therefore we refrain from referring these isolated elements beyond cf. *Axestemys splendida*.

## Institutional Abbreviations

KUVP: University of Kansas Natural History Museum and Biodiversity Institute, Lawrence, Kansas, USA
NCSM: North Carolina Museum of Natural Sciences, Raleigh, North Carolina, USA
PBMNH: The Palm Beach Museum of Natural History
ROM: Royal Ontario Museum, Toronto, Ontario, Canada
TMP: Royal Tyrrell Museum of Palaeontology, Drumheller, Alberta, Canada

## Additional Information and Declarations

### Data Availability

The following information was supplied regarding data availability: The research in this article did not generate any raw data.

## References

[ref-1] Brinkman DB (2003). A review of nonmarine turtles from the Late Cretaceous of Alberta. Canadian Journal of Earth Sciences.

[ref-2] Danilov IG, Hirayama R, Sukhanov VB, Suzuki S, Watabe M, Vitek NS (2014). Cretaceous soft-shelled turtles (Trionychidae) of Mongolia: new diversity, records and a revision. Journal of Systematic Palaeontology.

[ref-3] DePalma RA (2010). Geology, taphonomy, and paleoecology of a unique Upper Cretaceous bonebed near the Cretaceous-Tertiary boundary in South Dakota. MSc dissertation.

[ref-4] DePalma RA, Burnham DA, Martin LD, Larson PL, Bakker RT (2015). The first giant raptor (Theropoda: Dromaeosauridae) from the Hell Creek Formation. Paleontological Contributions.

[ref-5] Gardner JD, Russell AP, Brinkman DB (1995). Systematics and taxonomy of soft-shelled turtles (Family Trionychidae) from the Judith River Group (mid-Campanian) of North America. Canadian Journal of Earth Sciences.

[ref-6] Gilbert SF, Loredo GA, Brukman A, Burke AC (2001). Morphogenesis of the turtle shell: the development of a novel structure in turtle evolution. Evoloution and Development.

[ref-7] Hay OP (1908). Fossil turtles of North America.

[ref-8] Holroyd PA, Hutchison JH, Hartman JH, Johnson KR, Nichols DJ (2002). Patterns of geographic variation in latest Cretaceous vertebrates: evidence from the turtle component. The Hell Creek Formation and the Cretaceous-Tertiary boundary in the northern Great Plains: an integrated continental record of the end of the Cretaceous.

[ref-9] Holroyd PA, Wilson GP, Hutchison JH, Wilson GP, Clemens WA, Horner JR, Hartman JH (2014). Temporal changes within the latest Cretaceous and early Paleogene turtle faunas of northeastern Montana. Through the end of the Cretaceous in the type locality of the Hell Creek Formation in Montana and adjacent areas.

[ref-10] Hummel K (1928). Allgemeine Ergebnisse von Studien über fossile Weichschildkröten (Trionychia). Paläontologische Zeitschrift.

[ref-11] Hutchison JH, Brinkman DB, Holroyd PA, Gardner JD (2013). New turtles from the Paleogene of North America. Morphology and evolution of turtles: proceedings of the Gaffney Turtle Symposium (2009) in honor of Eugene S. Gaffney.

[ref-12] Hutchison JH, Archibald JD (1986). Diversity of turtles across the Cretaceous/ Tertiary boundary in northeastern Montana. Palaeogeography, Palaeoclimatology, Palaeoecology.

[ref-13] Nesbitt SJ, Turner AH, Spaulding M, Conrad JL, Norell MA (2009). The theropod furcula. Journal of Morphology.

[ref-14] Vickaryous MK, Hall BK (2010). Comparative development of the crocodylian interclavicle and avian furcula, with comments on the homology of dermal elements in the pectoral apparatus. Journal of Experimental Zoology Part B: Molecular and Development Evolution.

[ref-16] Vitek NS (2011). Insights into the taxonomy and systematics of North American Eocene soft-shelled turtles from a well-preserved specimen. Bulletin of the Peabody Museum of Natural History.

[ref-15] Vitek NS (2012). Giant fossil soft-shelled turtles of North America. Palaeontologia Electronica.

[ref-17] Vitek NS, Joyce WG (2015). A review of the fossil record of New World turtles of the clade Pan-Trionychidae. Bulletin of the Peabody Museum of Natural History.

